# Real-Time Sensor for Measuring the Surface Temperature of Thermal Protection Structures Based on the Full-Time Domain Temperature Inversion Method

**DOI:** 10.3390/s25072227

**Published:** 2025-04-02

**Authors:** Yuhao Liu, Xiong Zhao, Xiangyu Wei, Pengyu Nan, Fan Zhou, Guoguo Xin, Kok-Sing Lim, Yupeng Zhang, Hangzhou Yang

**Affiliations:** 1 School of Physics, Northwest University, Xi’an 710100, China; 202220846@stumail.nwu.edu.cn (Y.L.); 202210184@stumail.nwu.edu.cn (X.W.); xinguo@nwu.edu.cn (G.X.); 2 The 210 Institute of the Sixth Academy of CASIC, Xi’an 710065, China; zhaoxiong1208@163.com; 3 Innovation and Research Institute of HIWING Technology Academy, Beijing 100074, China; zhfa1010@163.com (F.Z.); zhangyupeng209@163.com (Y.Z.); 4 Photonics Research Centre, University of Malaya, Kuala Lumpur 500603, Malaysia; kslim@um.edu.my

**Keywords:** thermal protect structure, real-time sensor, IHCP, overlapping sliding window

## Abstract

**Highlights:**

**What are the main findings?**

- Real-time implementation of a full-time domain method (TIM-AB) is achieved by an overlapping sliding window method.
- The optimal window duration is equal to the thermal hysteresis time, and the effect of noise on the inversion accuracy can be effectively reduced by Savitzky-Golay filtering.

**What is the implication of the main finding?**

- The surface temperature of TPSs can be calculated by real-time inversion using the TIM-AB method.
- It is proved that the proposed method has great potential for engineering applications.

**Abstract:**

The real-time surface thermal monitoring of thermal protection structures (TPSs) is crucial for hypersonic vehicle safety. This study proposes an effective approach for real-time temperature reconstruction by integrating embedded sensor arrays with an enhanced full-time domain inversion algorithm, utilizing the overlapping sliding window method. An array of three evenly spaced sensors is used for TPS monitoring. Notably, the inversion approach eliminates the need for prior knowledge of the TPS’s thermal parameters. It exhibits remarkable practicality with low-frequency sampling requirements (1 Hz) and robust noise resistance. Through numerical simulations and a quartz lamp side heating experiment, it is demonstrated that the window size and data noise have great influence on the temperature reconstruction accuracy, but the window slip step has little influence. The mean relative error of the inversion temperature decreases exponentially as the window size increases, and the optimal window duration is equal to the thermal hysteresis time. The study investigates the impact of three noise filtering methods on the inversion accuracy, finding that the Savitzky-Golay filtering significantly enhances measurement precision, reducing mean relative error from 18.4% to 6.7%. These results highlight the potential of the proposed real-time sensor method for practical engineering applications, offering a reliable and efficient solution for real-time TPS temperature monitoring.

## 1. Introduction

In the aerospace field, an accurate and reliable temperature monitoring system is very important. The precise temperature monitoring of thermal protection structures (TPSs) is not only an essential foundation for the lightweight design and thermal insulation performance studies of these materials, but also a crucial basis for the safe operation and life health monitoring of hypersonic vehicle [1].

Conventional sensors are used to measure the internal temperatures of the TPS during various development phases, such as laboratory testing, benchmarking, and full-scale mockups. Researchers are continually working to improve sensor performance, with a focus on widening the temperature range, enhancing anti-interference capabilities, and broadening application scenarios [2,3,4]. However, the direct measurement of heat flux or surface temperature presents significant challenges. Common sensors, such as thermocouples, thermistors, and semiconductor sensors, are often susceptible to complex thermal load environments [5]. The operating environment for vehicles is extremely demanding, characterized by strong shear forces, exposure to extreme temperatures, and continuous vibration. These harsh conditions pose a significant challenge to the durability and reliability of sensors mounted externally [6,7,8]. Embedding sensors within the TPS can effectively enhance the survival rate of the sensors, and then, the surface temperature can be accurately reconstructed based on an inversion algorithm. The integration of sensors with various types of algorithms can effectively enhance the accuracy and stability of sensors, expand their functionalities, and even enable their application in new scenarios [9,10,11,12].

Accurately determining the surface temperature of a TPS from internal temperature measurements presents an inverse heat conduction problem (IHCP). Research efforts to address this challenge have primarily focused on two key methodologies: full-time domain inversion and sequential inversion. Full-time domain inversion is based on the whole temperature information to invert the unknown boundary over the entire time domain. Researchers solved the IHCP through various full-time domain inversion methods, including the Levenberg–Marquardt algorithm [13,14], Bayesian computation [15], Tikhonov regularization [16], transfer function methods [17], and spectral decomposition [18]. The full-time domain inversion method has proven to be effective in addressing IHCP, among which Tikhonov regularization stands out as a particularly effective approach [19]. It determines boundary heat flux or thermal physical parameters using an iterative optimization process guided by forward heat conduction model [20]. However, due to the inherent limitations of the full-time domain inversion method, this method relies on the accuracy of the forward problem solution. This implies that high computational resources are required to ensure both computational accuracy and high-speed iteration [21]. The classical full-time domain methods for solving IHCP face significant challenges in real-time implementation.

The sequential inversion method operates by estimating the boundary temperature or heat flux for the subsequent moment based on the results of the previous moment’s inversion. This process continues in a rolling manner until the unknown boundary information across the entire time domain is acquired. In other words, the sequential inversion is a real-time method. Different real-time inversion methods, including the filter form of Tikhonov regularization [22,23,24], the space marching method [25], Bayesian inference [26], Kalman filtering [27], and some improved methods based on neuron algorithm [28,29,30,31,32,33], have been proposed to solve inverse heat conduction problems in different engineering fields. By solving the IHCP problem in real time, the requirements of sensors in measuring the surface thermal information of TPSs can be effectively reduced.

A general iterative regularization method was used for the estimation of external heat flux for the thermal protection of a spacecraft by Nenarokomov et al. [7]. Uyanna et al. [22] developed a solution approach based on the filter form of the Tikhonov regularization method to calculate the surface heat flux in an integrated thermal protection system in real time. Qi et al. [27] introduced the Kalman filtering (KF) algorithm to address the inverse coupled radiation–conduction heat transfer problem in a participating medium. They analyzed the impact of various factors, such as measurement noise and processing noise, on the accuracy and stability of the KF algorithm. Nakamura et. al. [34] developed a computational method of transient inverse heat conduction analysis for applying to problems of two-dimensional plate and atmospheric re-entry capsule. Woodbury et al. [35] proposed a method for calculating the thermal action at the remote boundary using a second measured temperature history of points inside the domain. Huang et al. [36] proposed a real-time solution method for improving the whale optimization algorithm and a parameter-adaptive proportional integral derivative controller to solve the problem of common unsteady IHCP in the industrial field. Najafi et al. [37] estimated the multiple unknown heat fluxes at the bottom of a plate by solving a real-time solution for a two-dimensional inverse heat conduction problem. Deng et al. [38] combined Hopfield neural networks with BP neural networks to solve the heat conduction inverse problem, identifying unknown boundary conditions.

Our team has proposed a novel full-time domain temperature inversion method [39]. It is called the temperature inversion method with adaptive boundary (TIM-AB), which is based on Fourier’s law of heat conduction and combined with the Auto-Regression with eXtra (ARX) data processing technique. The TIM-AB method stands out for its ability to precisely evaluate the surface temperature of TPSs by utilizing temperature measurements from three distinct points within the material and does not require knowledge of the thermal parameters of the TPS. Although this method is a full-time domain inversion approach, it can still rapidly achieve temperature inversion without relying on high performance computing devices. Therefore, this method allows the real-time temperature determination of TPS.

In this paper, the real-time implementation of the TIM-AB method is demonstrated. The sliding window overlapping sampling method is used in real-time operations. Through numerical simulations and a lateral heating experimental system, we analyze the impact of the moving window size and sensor data sampling frequency on the accuracy of real-time inversion. Furthermore, through quartz lamp lateral heating experiments on TPSs, the impact of thermocouple noise under strong electromagnetic interference on real-time inversion is analyzed. An effective noise-filtering method is adopted, which can significantly reduce the influence of noise. The TIM-AB method is a promising solution for the real-time temperature inversion model for various engineering applications.

## 2. Real-Time Implementation of TIM-AB

### 2.1. The Temperature Inversion Method with Adaptive Boundary

**Figure 1:**
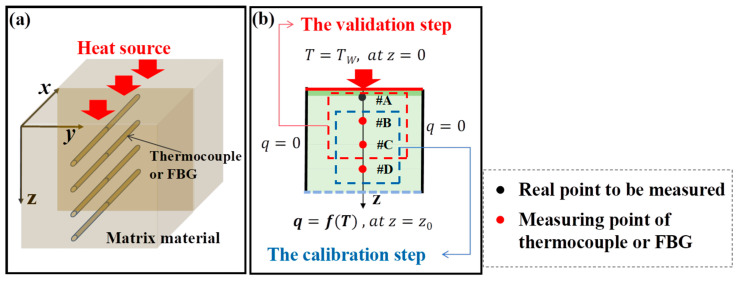
(**a**) Schematic diagram of the sensors (thermocouple or FBG) embedded in the TPS, and (**b**) illustrative diagram of the surface temperature estimation by the TIM-AB method.

The sensor arrangement when using the TIM-AB method is shown in Figure 1a. When the surface temperature or heat flux of the TPS is relatively uniform, the heat conduction process along the thickness direction can be approximated to quasi-one-dimensional heat conduction, as shown in Figure 1b. On both sides of the material, the boundary conditions are adiabatic, *q* = 0. The environment of the lower surface of the material is arbitrary, *q* = *f*(*T*), at z = z_0_. Points *#A*, *#B*, *#C*, and *#D* are equidistant along the thickness direction. Firstly, the heat transfer relationship between points *#B*, *#C*, and *#D* is calibrated by the full-time domain temperature data. Secondly, this relation is applied to points *#A*, *#B*, and *#C*, so that the temperature at point *#A* is calculated based on the temperature at points *#B* and *#C*.

In the calibration step, the heat transfer relationship between *#B*, *#C*, and *#D* can be described using a matrix form [39].

(1) $$\hat{A}=\hat{B}\hat{X}$$

where

(2) $$\hat{A}={\left[{\left(T_{B}\right)}_{t=\delta t}{\left(T_{B}\right)}_{t=2\delta t}\cdots {\left(T_{B}\right)}_{t=n\delta t}\right]}^{T}$$

and

(3) $$\hat{B}=\left[\begin{array}{ccc} {\left.\frac{dT_{C}}{dt}\right|}_{t=\delta t} & {\left(T_{C}\right)}_{t=\delta t} & -{\left(T_{D}\right)}_{t=\delta t}\\ {\left.\frac{dT_{C}}{dt}\right|}_{t=2\delta t} & {\left(T_{C}\right)}_{t=2\delta t} & -{\left(T_{D}\right)}_{t=2\delta t}\\ \vdots & \vdots & \vdots \\ {\left.\frac{dT_{C}}{dt}\right|}_{t=n\delta t} & {\left(T_{C}\right)}_{t=n\delta t} & -{\left(T_{D}\right)}_{t=n\delta t} \end{array}\right]$$

where *T_B_*, *T_C_*, and *T_D_* represent the temperatures of *#B*, *#C*, and *#D*, respectively. *δt* represents the time interval at which the sensor collects data. *n* indicates the total number of data collected during the entire collection period.

$\hat{X}$ is the coefficient matrix, which can be written as

(4) $$\hat{X}=\begin{array}{ccc} {}[\hat{\tau ’} & \hat{\xi ’}+1 & \hat{\xi ’}{]}^{T} \end{array}$$

$\hat{X}$ can be calculated using the least-squares method.

(5) $$\hat{X}={\left(\hat{B^{T}}\hat{B}\right)}^{-1}\hat{B^{T}}\hat{A}$$

In the validation step, when the thermal properties of the matrix material over the region of these four points are uniform and stable, two sets of data of *#A*, *#B*, *#C* and *#B*, *#C*, *#D* share the same coefficient matrix [39]. That is,

(6) $$\begin{array}{c} \hat{X}=X’\\ \begin{array}{ccc} {}[\overset{\Lambda }{\tau }’ & \overset{\Lambda }{\xi }’+1 & \overset{\Lambda }{\xi }’{]}^{T} \end{array}=\begin{array}{ccc} {}[\tau ’ & \xi ’+1 & \xi ’{]}^{T} \end{array} \end{array}$$

The coefficient matrix X’ describes the heat transfer relationship between *#A*, *#B*, and *#C*.

(7) $$X’={\left(B{’}^{T}B’\right)}^{-1}B’A’$$

where

(8) $$A’={\left[{\left(T_{A}\right)}_{t=\delta t}{\left(T_{A}\right)}_{t=2\delta t}\cdots {\left(T_{A}\right)}_{t=n\delta t}\right]}^{T}$$

and

(9) $$B’=\left[\begin{array}{ccc} {\left.\frac{dT_{B}}{dt}\right|}_{t=\delta t} & {\left(T_{B}\right)}_{t=\delta t} & -{\left(T_{C}\right)}_{t=\delta t}\\ {\left.\frac{dT_{B}}{dt}\right|}_{t=2\delta t} & {\left(T_{B}\right)}_{t=2\delta t} & -{\left(T_{C}\right)}_{t=2\delta t}\\ \vdots & \vdots & \vdots \\ {\left.\frac{dT_{B}}{dt}\right|}_{t=n\delta t} & {\left(T_{B}\right)}_{t=n\delta t} & -{\left(T_{C}\right)}_{t=n\delta t} \end{array}\right]$$

The reconstruction temperature ${\hat{T}}_{A}$ at point *#A* can be estimated using the following equation.

(10) $$\hat{T_{A}^{n}}=\hat{\tau ’}{\left.\frac{\partial \hat{T_{B}}}{\partial t}\right|}_{t_{n}}+(\hat{\xi ’}+1)\hat{T_{B}^{n}}-\hat{\xi ’}\hat{T_{C}^{n}}$$

That is,

(11) $$A’=B’\hat{X}$$

### 2.2. The Implementation Method of Real-Time Inversion

The full-time domain inversion method requires a minimum amount of data before the inversion calculation can begin. This process of accumulating data is known as data buffering, and the time required for buffering is defined as the window size. Once data buffering is complete, the inversion calculation is performed using both the accumulated data and the newly acquired data. In subsequent iterations, the calculation continues with the same window size, shifting progressively to the next position. This process is repeated until data acquisition is complete. This approach is known as the sliding window overlapping sampling method. Data buffering determines the algorithm’s initialization time, while the computation time within a single window dictates the delay in real-time inversion. The moving window size is a crucial parameter—an excessively large window increases computational delay, whereas a smaller window may degrade inversion accuracy.

Based on the temperature relationship (Equation (11)) between the four points of *#A*, *#B*, *#C*, and *#D*, the window function is defined as

(12) $$A^{’}(i,j)=B^{’}(i,j)\hat{X}(i,j)$$

where *i* and *j* represent the start and end of the data, respectively.

The window size is denoted as *t_w_*. The amount of data in one window period is *n_w_*, *n_w_* = *t_w_ f_s_*. *f_s_* is the sampling frequency of the sensor, $f_{s}=1/\delta_{t}$. Therefore, there is the relationship, *j* = *i* + *n_w_* − 1, 1 ≤ *i* ≤ *j* ≤ *n*. *n* is the total number of data in the full-time domain.

Given the characteristics of the experimental data in this study that comprise both uniformly distributed small-range data (constant temperature data) and highly heteroscedastic data (instantaneous temperature rise data), the mean relative error was employed to assess the accuracy of the inversion results, with particular emphasis on the inversion of instantaneous temperature rise data. The mean relative error between the inversion results A’(i,j) and the real results $\overset{\sim }{A}(i,j)$ in a time window is defined as the objective function.

(13) $${\bar{\delta }}_{w}=\bar{\delta }\left(i,j\right)=\frac{1}{n_{w}}\sum_{k=i}^{i+n_{w}-1}\frac{\left|{\left(T{’}_{A}\right)}_{t=k\delta t}-\tilde{(T_{A}}{)}_{t=k\delta t}\right|}{\tilde{(T_{A}}{)}_{t=k\delta t}}$$

The optimal value of the sliding window was chosen to minimize the objective function. The optimal value within a single window was found.

(14) $$min\left(\bar{\delta }(i,i+n_{w}-1)\right)$$

The optimal value within full-time domain was found.

(15) $$min\left(\bar{\delta }=\frac{1}{\frac{n-n_{w}}{n_{s}}+1}\sum_{i=1}^{\left(\frac{n-n_{w}}{n_{s}}+1\right)\cdot n_{s}+1}\bar{\delta }\left(i,j\right)\right)$$

where n_s_ is the sliding step of window.

Complex information can often be decomposed by using Gaussian decomposition [40,41]. The thermal loads can be decomposed into a series of Gaussian thermal pulses. Taking the Gaussian pulse thermal shock as a typical heat source, the optimal value of window size in inversion calculation was analyzed. Gaussian pulse thermal shock can be expressed as

(16) $$q(t)=q_{0}e^{\left(-\frac{{\left(t-2\tau ’\right)}^{2}}{2\tau {’}^{2}}\right)}$$

The standard variance of Gaussian thermal shock was set as the thermal hysteresis time τ’ of the TIM-AB method, $\tau ’=\frac{\rho c}{2k}z_{AB}\left(z_{AB}+z_{BC}\right)$. The window size coefficient η was defined to reflect the relationship between the window time *t_w_* and the thermal hysteresis time τ’. That is, $t_{w}=\eta \tau ’$, $n_{w}=t_{w}f_{s}=\eta \tau ’f_{s}$.

## 3. Feasibility Analysis of Real-Time Inversion

### 3.1. Surface Temperature Estimation by TIM-AB Method

**Figure 2:**
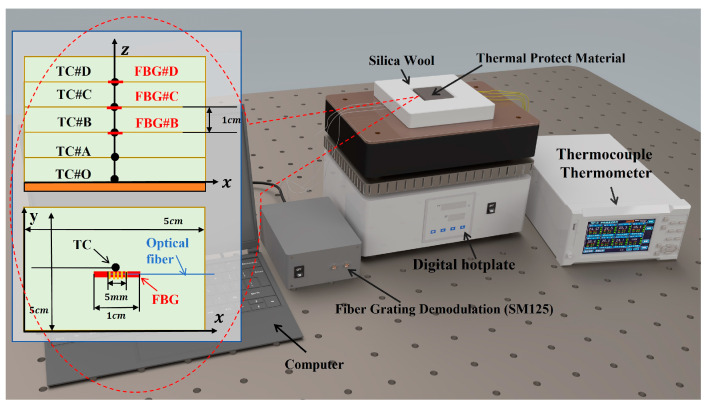
The experimental equipment for lateral heating of the thermal insulation material.

The experimental equipment for the lateral heating of thermal insulation material is shown in Figure 2. The bottom of the thermal insulation material was heated by a digital heating hotplate. The upper surface was set to natural convection. The other sides were wrapped by a heat-insulating silica wool to form an adiabatic condition. The size of thermal insulation material was 5 cm × 5 cm × 5 cm. The temperatures at points *#A*, *#B*, *#C*, and *#D* inside the material were measured by thermocouple (TC) and fiber Bragg grating (FBG) sensors, as illustrated in Figure 2. The K-type thermocouple had a probe size of ~0.3 mm and a response delay of ~100 ms. Each FBG was encapsulated and protected in a 0.5 mm diameter corundum tube. Point *#O* is located at the contact surface between the material and the hotplate. Due to the complexity and instability of the heat transfer mechanism of the contact surface, the temperature at point *#O* that indicates the bottom temperature was monitored by a TC.

**Figure 3:**
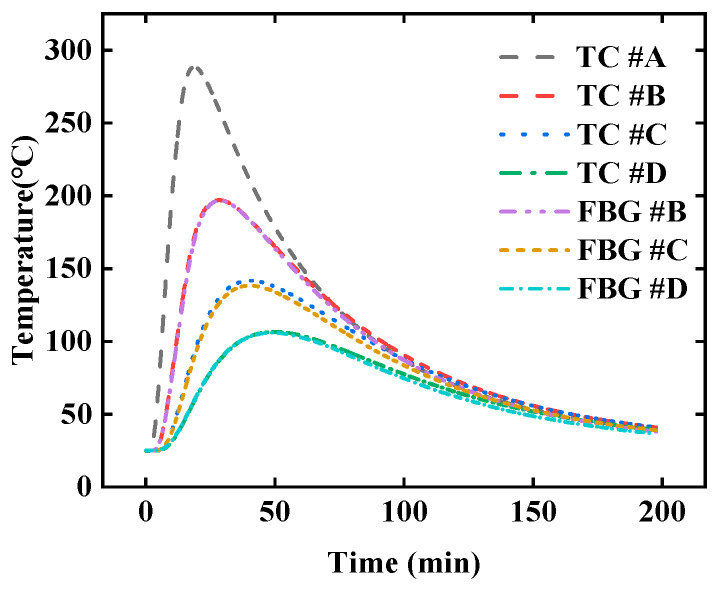
The temperature curves at different positions measured by FBG and TC.

The temperature at point *#A* was selected as the target of the inversion, and the temperature at points of *#B*, *#C*, and *#D* were the input data of the inversion. The comparison of the temperature measured by TC and FBG is shown in Figure 3. The temperature measurement results of FBG and TC have little deviation. If it is only for measuring the internal temperature of the material, the temperature deviation of the two sensors can be ignored.

**Figure 4:**
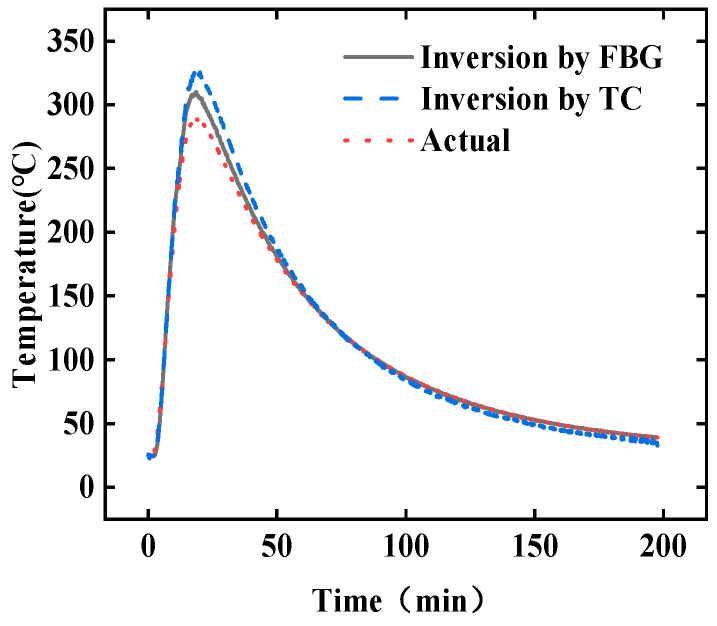
The inversion temperature at point *#A* calculated based on FBG and TC data.

**Figure 5:**
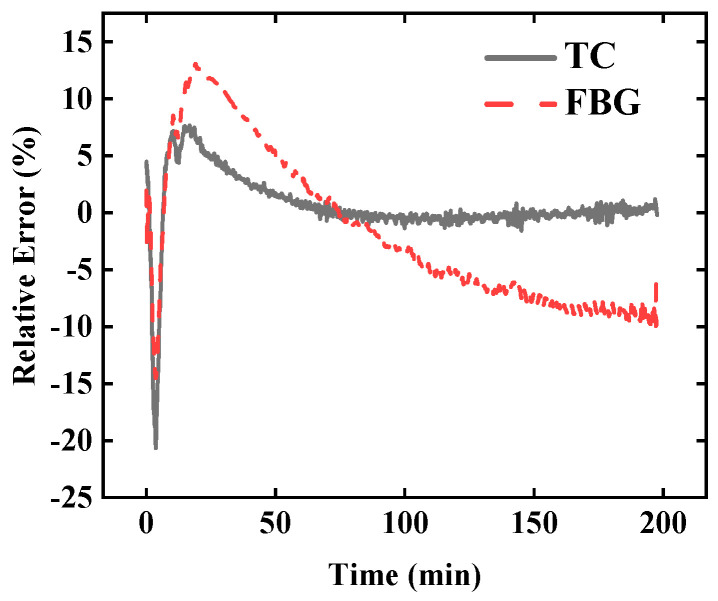
The relative error of the inversion temperature at point *#A* based on FBG and TC data.

Based on the FBG data and the TC data respectively, the inversion temperature at point *#A* calculated by the TIM-AB method is as shown in Figure 4, and the relative error of the inversion temperature is shown in Figure 5. The temperature measured by the TC is taken as the actual reading at point *#A*. The mean relative error for the TC data is less than 1.6%, whereas the mean relative error for FBG data is less than 6.2%. Both measurement tools ensure the high accuracy of the algorithm the error based on TC is smaller. If the inversion accuracy based on FBG is to be improved, the thermal hysteresis correction algorithm of the tube-packaged FBG sensor can be used, as detailed in Ref. [39].

### 3.2. The Relationship Between Moving Window Size and the Thermal Hysteresis Time

**Figure 6:**
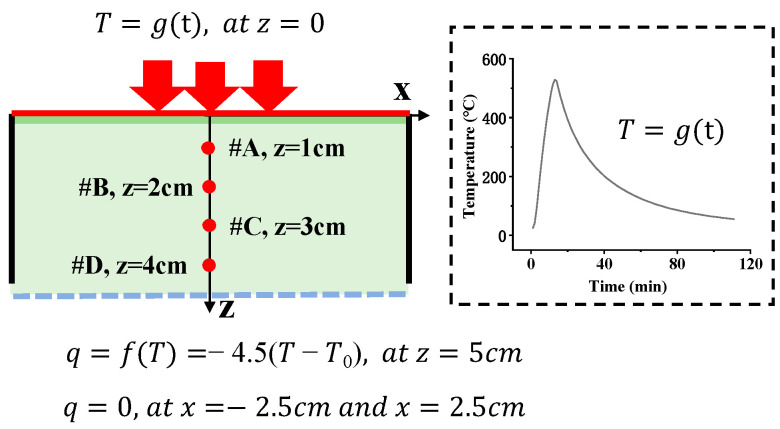
Schematic diagram of the quasi-one-dimensional heat conduction model under lateral heating.

A quasi-one-dimensional heat conduction finite element model was established to simulate the lateral heating experiment of the thermal insulation material. As shown in Figure 6, one end of the material is a heat source, with its temperature variation determined by the experimentally measured temperature data at point *#O*. The other end was subjected to convective cooling with a boundary condition defined as *q* = −4.5(T − T_0_) W/m^2^. Both lateral sides were adiabatic boundaries. The density ρ, specific heat capacity *c*, and thermal conductivity *k* were 350 Kg/m^3^, 1200 J/Kg/K, and 0.09 W/m/K, respectively.

**Figure 7:**
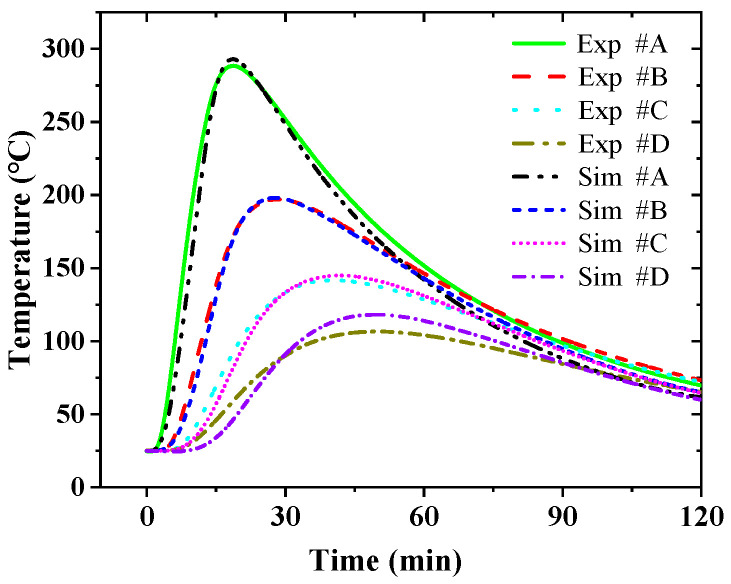
Comparison of the numerical simulation results and experimental results of the temperature at different points in the thermal insulation material.

The simulated results of the temperature at points *#A*, *#B*, *#C*, and *#D* were compared to those measured by the TC, as shown in Figure 7. The results show that the numerical simulation results are basically consistent with the experimental results. Although the heat transfer mechanism inside the thermal insulation material is very complex, the heat transfer process can be simplified to the classical Fourier heat transfer process under the long-term loading of small heat flow. Therefore, the temperature evolution under Gaussian pulse thermal shock was analyzed by numerical simulation, and then the effect of the window size on the accuracy of real-time inversion was analyzed.

**Figure 8:**
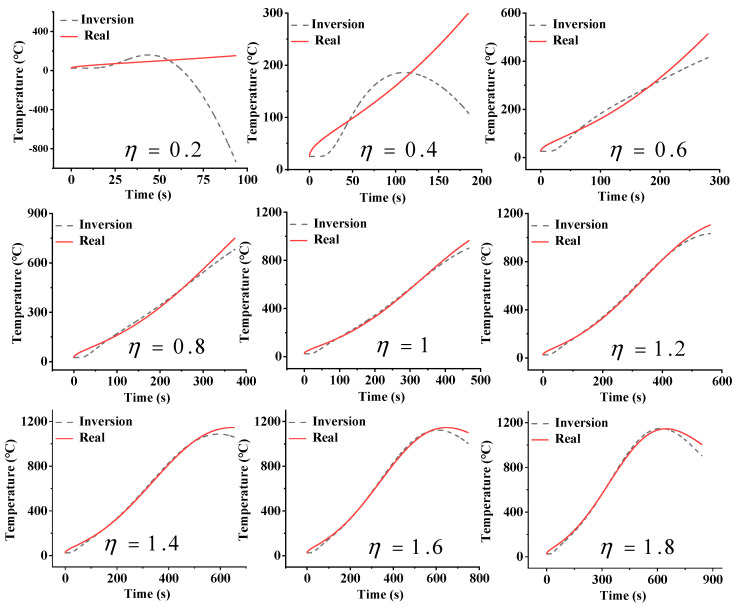
The inversion temperature at point *#A* using different window sizes, when the sampling frequency *f_s_* is 100 Hz.

**Figure 9:**
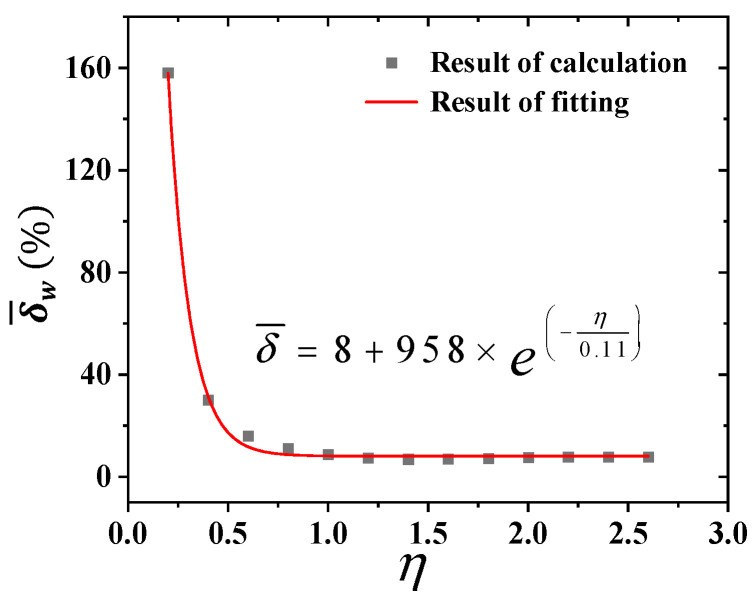
The relationship between the mean relative error ${\bar{\delta }}_{w}$ of the inversion results and the window size coefficient η, when the sampling frequency *f_s_* is 100 Hz.

In numerical simulation, the Gaussian pulse thermal shock $q(t)=q_{0}\exp(-\frac{{\left(t-2\tau ’\right)}^{2}}{2\tau {’}^{2}})$, $\tau ’=\frac{\rho c}{2k}z_{AB}\left(z_{AB}+z_{BC}\right)=467s$. *q*_0_ is the peak power density, 1 W/cm^2^. Z_AB_ is the distance between points *#A* and *#B*. Z_BC_ is the distance between points *#B* and *#C*. Figure 8 presents the inversion temperature responses at point *#A* for differently adopted window sizes, $t_{w}=\eta \tau ’$. The sampling frequency *f_s_* is 100 Hz. When the window size coefficient η is less than 0.4, it is completely impossible to calculate the temperature at point *#A* through the inversion method, and the inversion results have no correlation with the actual results. In this case, the duration of the measurement is very short, much shorter than the thermal hysteresis time. The thermocouple (point *#B*) closest to the heat source cannot effectively detect the changes in the external thermal load. The thermocouples (points *#C* and *#D*) far from the heat source are even less capable of obtaining valid information. Therefore, it is difficult to invert the external temperature from the internal temperature data. When η is greater than 0.6, the inversion results closely approximate the actual results. Figure 9 shows the relationship between the mean relative error ${\bar{\delta }}_{w}$ of the inversion results and η. When η = 0.2, ${\bar{\delta }}_{w}$ is close to 160%. When η = 0.4, ${\bar{\delta }}_{w}$ quickly drops to 30%, and when η = 1, ${\bar{\delta }}_{w}$ gradually stabilizes to about 8%. ${\bar{\delta }}_{w}$ decreases exponentially with the increase in η. The fitted exponential function is ${\bar{\delta }}_{w}=8+958\exp(-\eta /0.11)$.

**Figure 10:**
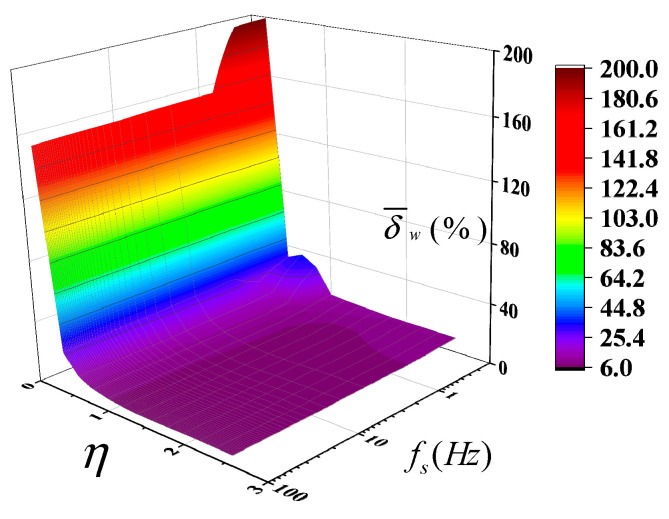
The mean relative error ${\bar{\delta }}_{w}$ of the inversion results at different window sizes and sampling frequencies.

Figure 10 presents the mean relative error ${\bar{\delta }}_{w}$ of the inversion results at different window sizes and sampling frequencies. ${\bar{\delta }}_{w}$ is basically exponential with η at different sampling frequencies *f_s_*. When *f_s_* is less than 1 Hz, ${\bar{\delta }}_{w}$ becomes significantly larger. At η = 0.2, ${\bar{\delta }}_{w}$ reaches 200%. Furthermore, the relationship between ${\bar{\delta }}_{w}$ and η deviates from an ideal exponential curve, displaying oscillatory behavior at η = 1. When η reaches 1.6, ${\bar{\delta }}_{w}$ converges to a constant value of ~10%. When *f_s_* exceeds 1 Hz, ${\bar{\delta }}_{w}$ consistently follows an exponential relationship with η. Even when *f_s_* increases from 1 Hz to 100 Hz, the maximum mean relative error only shifts from 158% to 156%, and the minimum decreases from 7.8% to 6.4%. Therefore, ${\bar{\delta }}_{w}$ is highly sensitive to variations in η but remains relatively insensitive to the sampling frequency during inversion calculations.

It should be noted that the inversion calculation method fundamentally involves determining the undetermined coefficients of the temperature distribution function derived from heat conduction theory through the least-squares method. Theoretically, its accuracy is governed by both the time derivative of the temperature and the data volume. Consequently, the observed insensitivity of the mean relative error to the sampling frequency in Figure 10 arises because the thermal insulation material exhibits a large thermal hysteresis time. This ensures that a sufficient window data sample size *n*_*w*_ is maintained, even at lower sampling frequencies, to ensure robust inversion calculations.

### 3.3. The Influence of the Slide Step of Window

The impact of the slide step of window *n_s_* on the mean relative error $\bar{\delta }$ is minimal. The numerical simulations reveal that, when the sampling frequency *f_s_* of the data is set at 10 Hz, increasing n_s_ from 1 to 10 has virtually no effect on the mean relative error, which remains steady at approximately 5.79%. Even when *n_s_* is further increased to 20, the mean relative error still exhibits negligible change. This stability can be attributed to the fact that the proportion of sliding data relative to the total amount of data within a window is exceedingly small. Specifically, *n_s_*/*n_w_* = 10/(467 × 10) × 100% = 0.21%.

While n_s_ has minimal impact on the accuracy of real-time inversion, it directly influences the output format and delay of the inversion results. When n_s_ = 1, the sensor acquisition frequency aligns with the output data frequency after algorithm processing, with the delay primarily arising from computation. In contrast, when ns = 10, the algorithm processes and outputs 10 data points collected within one second in a single computation. Although the output data frequency remains consistent with the data collection frequency, the delay increases significantly due to both data accumulation and processing time.

## 4. Real-Time Inversion of Surface Temperature of the TPS Under Transient Thermal Shock

### 4.1. Experimental Method

**Figure 11:**
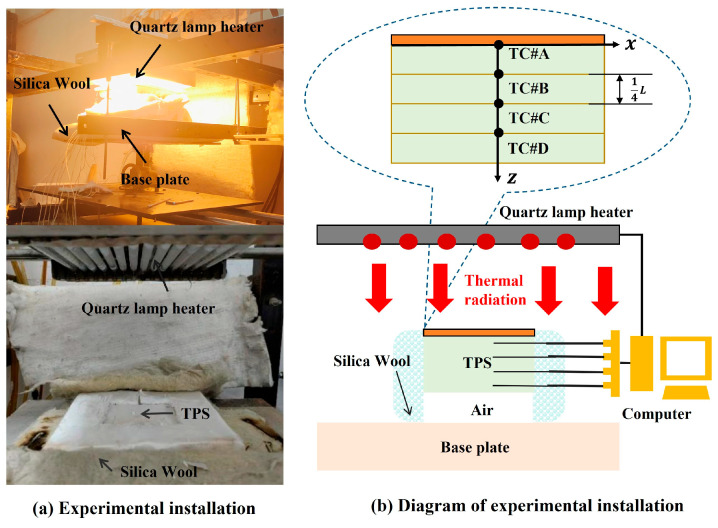
The experimental equipment for the lateral heating of the TPS using the quartz lamp heater, (**a**) experimental installation, (**b**) diagram of experimental instruction.

The experimental setup for the thermal characterization of the TPS is as shown in Figure 11. The setup mainly comprised a quartz lamp, base plate, heat insulation cotton, and data acquisition and processing. The upper surface of the TPS serves as the radiation surface, while the lower surface subjects to natural convection. A natural convection environment was established by elevating the TPS, thereby enabling the lower surface to be fully exposed to the air. With the exception of the top and bottom surfaces, the remaining sides of the TPS were encapsulated with heat-insulating silica wool, ensuring an approximately adiabatic condition. Inside the TPS sample, a set of TC was arranged along the z-axis (See Figure 11b). The K-type TC had a probe size of ~0.5 mm and a response delay of ~250 ms. The thermocouple sampling frequency was 10 Hz. The four thermocouples at *#A*, *#B*, *#C*, and *#D* were equidistant. The effective thickness of the material was *L*, and the distance between the two measuring points was *L*/4.

### 4.2. Comparison of Full-Time Inversion and Real-Time Inversion

**Figure 12:**
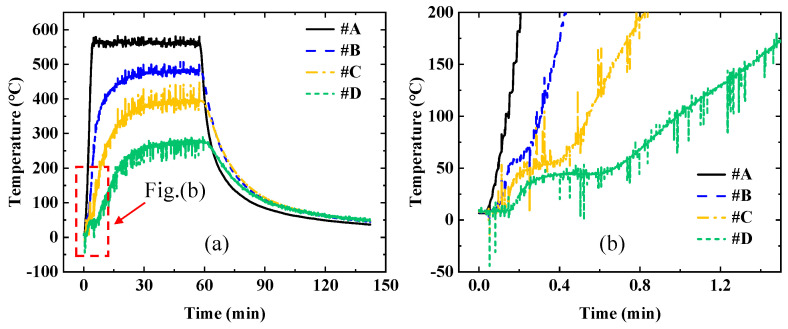
The temperature variation curves of points *#A*, *#B*, *#C*, and *#D* under quartz lamp heating, (**a**) the temperature of the entire heating process, (**b**) the temperature at the initial moment of heating.

The temperature variation curves of points *#A*, *#B*, *#C*, and *#D* under quartz lamp heating are shown in Figure 12. The surface temperature of the TPS rapidly increases at the instant of quartz lamp irradiation, forming a distinct temperature gradient within the material. During the operation of the quartz lamp, significant electromagnetic interference is generated, resulting in substantial noise in the temperature data collected by TC. In real application scenarios, TPSs are also subjected to complex electromagnetic interference, which lead to inaccurate temperature measurements. Therefore, it is crucial to analyze the sensitivity of the inversion algorithm to noise and effectively eliminate its impact.

TPSs consist of different types of porous materials. Their internal heat transfer mechanism is highly complex, involving radiative heat transfer, convective heat transfer, and solid heat transfer [42,43,44,45]. During the brief period of quartz lamp activation, radiative heat transfer accounts for a significant proportion of the heat transfer within the material, and a certain thickness of the material is heated simultaneously, as shown in Figure 12b. Non-solid heat transfer modes can reduce the accuracy of the inversion algorithm.

**Figure 13:**
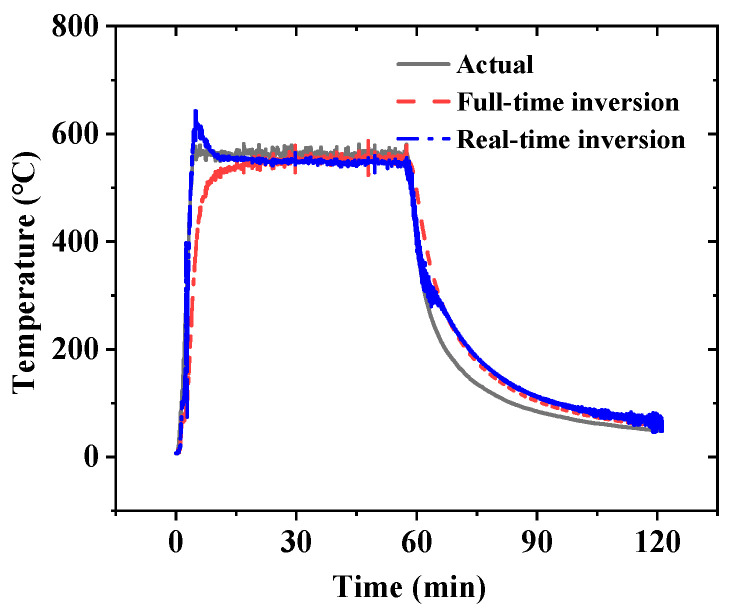
The comparison between the full-time domain inversion temperature, the real-time inversion temperature, and actual temperature at point *#A*.

Without noise filtering, the temperature at point *#A* was calculated using both the full-time inversion method and the real-time inversion method. The comparison between the inversion temperatures from these two methods and the true temperature at point *#A* is shown in Figure 13. The real-time inversion method used a window size of η = 1. The results demonstrate that, under real test conditions, the real-time inversion method effectively calculates the material’s surface temperature in real time. Compared to the full-time domain inversion results, the real-time inversion exhibits overshoot during the instantaneous temperature rise phase. In full-time domain inversion, this phenomenon may also occur if the sensor collects insufficient data during the instantaneous temperature rise, leading to an unclear temperature transfer relationship between measurement points [39]. During the temperature-holding phase, the results from the full-time domain inversion are closer to the true values. In the first half of the cooling phase, the results from the real-time inversion method are closer to the true values. In the second half of the cooling phase, the full-time inversion method has a slightly higher accuracy than the real-time inversion method. Overall, the mean relative error $\bar{\delta }$ of the results from the full-time domain inversion method is approximately 15.7%. The results calculated using the real-time inversion method contain more noise, with a mean relative error $\bar{\delta }$ of about 18.8%.

**Figure 14:**
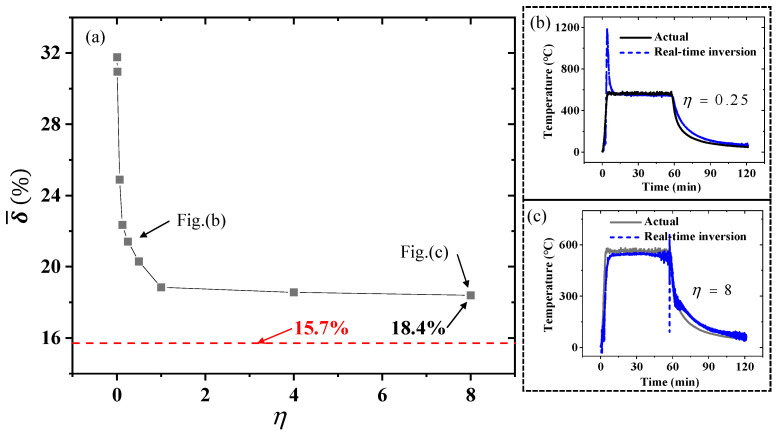
The accuracy of the real-time inversion method using different window sizes. (**a**) The relationship between the mean relative error $\bar{\delta }$ and the window size coefficient η; (**b**) the comparison of real-time inversion temperature and real temperature, when η is 0.25; and (**c**) the comparison of real-time inversion temperature and real temperature, when η is 8.

The relationship between the accuracy of the real-time inversion method and the window size is shown in Figure 14. The mean relative error $\bar{\delta }$ of the inversion results has an exponential relationship with the window size coefficient η, which is consistent with the numerical simulation results. When η is very small, the error in the inversion results is very large, and the error is mainly concentrated in the instantaneous heating phase and the cooling phase. When η is greater than 1, the inversion error decreases very slowly with the increase in η. As η increases from 1 to 8, $\bar{\delta }$ decreases from 18.8% to 18.4%.

Comparing Figure 13 and Figure 14c, it can be seen that, although the difference in the mean relative error is not significant, the distribution of errors is different. When η is 8, the error in the heating phase decreases significantly, while the error in the cooling phase becomes more noticeable and the noise in the inversion results increases. It can be predicted that, if η increases to 563, which is equivalent to covering the entire data with one window (the total data volume is approximately 563 × 1280), the real-time inversion will degenerate into full-time domain inversion, and the inversion error will decrease to 15.7%.

### 4.3. The Influence of Measurement Uncertainty on Real-Time Inversion

The measurement uncertainty of the sensor is a factor worthy of analysis. Given the limited sample size in the measurement process, we considered only the K-type thermocouple itself and measuring equipment. The type B evaluation method was used to calculate the standard uncertainty components, followed by the calculation of the combined standard uncertainty and expanded uncertainty. When the temperature measurement range was within 600 °C, the measurement uncertainty of the K-type thermocouple was ~±0.56 °C.

To analyze the impact of thermocouple measurement uncertainty on real-time inversion results, we introduced normally distributed random errors to the measured temperatures at points *#A*, *#B*, *#C*, and *#D*. The random errors ranged from −0.56 °C to 0.56 °C. The real-time inversion temperature of point A was obtained using the temperature data from points *#B*, *#C*, and *#D* that incorporated these uncertainty errors. The entire calculation process involved approximately 7200 iterations implemented by moving window method. As each temperature measurement receives randomly generated errors, this setup effectively provide sufficient repeated sampling for statistical significance.

**Figure 15:**
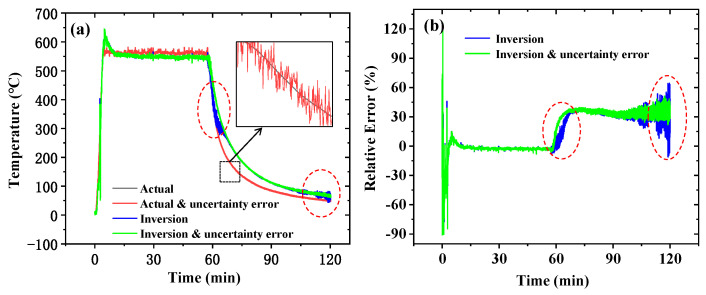
The influence of measurement uncertainty on real-time inversion. (**a**) A comparison of actual data, modified data with random error, inversion results using actual data, and inversion results using error-containing data; (**b**) a comparison of the relative error of inversion results from data with and without random errors.

Figure 15a displays four temperature profiles about point *#A*: the actual temperature; the modified temperature with random errors (actual and uncertainty error); the inversion temperature using the original data from *#B*, *#C*, and *#D*; and the inversion temperature using error-containing data. Figure 15b compares the relative errors between inversions using actual data versus error-containing data. The results demonstrate that measurement uncertainties affect inversion accuracy, particularly during the initial cooling phase and the temperature stabilization phase. While measurement errors amplified inversion errors during cooling, they exhibited error-reducing effects during temperature stabilization. Statistical analysis reveals the mean relative error increased from 18.8% (using actual data) to 20.6% when accounting for measurement uncertainties.

### 4.4. The Influence of Data Noise on the Real-Time Inversion

**Figure 16:**
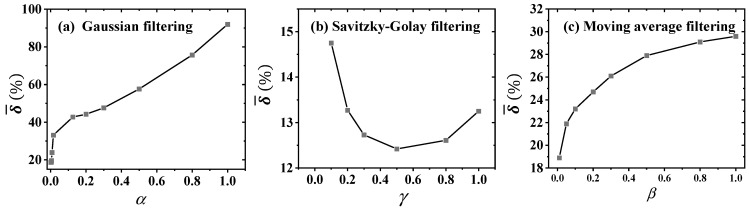
The effects of three typical noise filtering methods, when the window size coefficient η = 1 was used for the real-time inversion. (**a**) The change in mean relative error $\bar{\delta }$ with the proportionality coefficient α by Gaussian noise filtering, (**b**) the change in mean relative error $\bar{\delta }$ with the proportionality coefficient γ by Savitzky-Golay noise filtering, and (**c**) the change in mean relative error $\bar{\delta }$ with the proportionality coefficient β by moving average noise filtering.

The effects of three typical noise filtering methods, Gaussian noise filtering, Savitzky-Golay noise filtering, and moving average noise filtering, are shown in Figure 16. The window size coefficient η = 1 was used for the real-time inversion. Figure 16a shows the effect of Gaussian noise filtering. α represents the ratio of *n_G_* to the window size for real-time inversion. *n_G_* is the number of non-zero points. *n_G_* = 1 indicates no noise filtering. As α increases, the mean relative error $\bar{\delta }$ of the inversion temperature increases monotonically. Figure 16b shows the effect of Savitzky-Golay noise filtering. The polynomial order was set to 3. γ represents the ratio of the window size for Savitzky-Golay noise filtering to the window size for real-time inversion. As γ increases, $\bar{\delta }$ first decreases and then increases. $\bar{\delta }$ is minimized when γ = 0.5. Figure 16c shows the results of moving average noise filtering. β represents the ratio of the window size for moving average noise filtering to the window size for real-time inversion. $\bar{\delta }$ gradually increases as β increases.

Of the three noise filtering methods, the Savitzky-Golay noise filtering method performs relatively better and can reduce the error to a certain extent. The other two noise filtering methods fail to reduce the error in the real-time inversion. This is mainly because the real-time implementation method of TIM-AB proposed in this paper is highly sensitive to the time derivative of the data. Both Gaussian noise filtering and moving average noise filtering essentially lose data details, which can easily amplify the error in the inversion. Especially during real-time inversion, the amount of data available for each iteration is very limited. Noise filtering methods such as data averaging can cause more severe loss of data details and even destroy the regularity of the limited data. During full-time domain inversion, due to the large amount of data, the appropriate average data noise filtering can also preserve the overall law of the data, so as to reduce the error on the whole.

A further analysis was conducted on the influence of the noise filtering window size of the Savitzky-Golay noise filtering method on the inversion accuracy. Two settings were considered:

**Variable Noise Filtering Window Size:** The noise filtering window size was set to half of the real-time inversion window size, γ = 0.5. This means the noise filtering window size will change in real time with the inversion window size.

**Fixed Noise Filtering Window Size:** The noise filtering window size was set to a fixed value, equivalent to 0.1 times the thermal hysteresis time, that is, γ = 0.1/η. When the inversion window size is small (η < 0.1), γ > 1. Since the theoretical maximum value of γ is 1, the noise filtering window size is forcibly set to match the real-time inversion window size.

**Figure 17:**
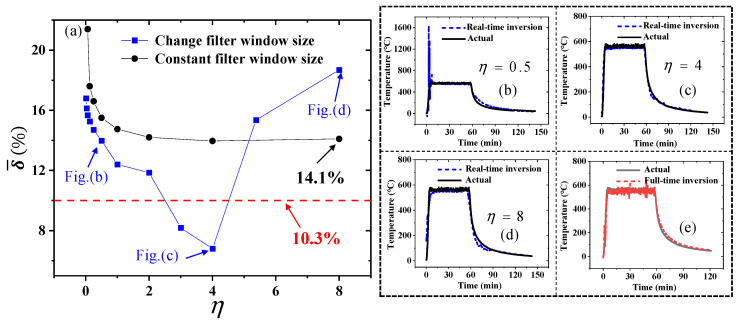
(**a**) Under two window size settings of Savity–Golay filtering, the mean relative error varies with the window size of real-time inversion; (**b**) the comparison of the inversion temperature with the true temperature, when η = 0.5; (**c**) the comparison of the inversion temperature with the true temperature, η = 4; (**d**) the comparison of the inversion temperature with the true temperature, η = 8; and (**e**) the temperature calculated by full-time domain inversion method based on the denoised global data.

After applying these two settings for noise filtering, the results are shown in Figure 17a. The results indicate that, when the noise filtering window size is constant, the mean relative error $\bar{\delta }$ still has an exponential relationship with the inversion window size coefficient η. Noise filtering can effectively reduce inversion errors, with $\bar{\delta }$ decreasing from 18.8% before noise filtering to 14.1% after noise filtering. However, this is still higher than the accuracy of full-time domain inversion after noise filtering, which has an mean relative error of 10.3%.

When the noise filtering window size is always half of the real-time inversion window size, the mean relative error $\bar{\delta }$ shows instability. When η ≤ 2, an exponential relationship between $\bar{\delta }$ and η can still be observed. However, as η increases, $\bar{\delta }$ rapidly decreases and then rapidly increases again. At η = 4, $\bar{\delta }$ reaches its minimum, approximately 6.7%, which is already lower than the error of full-time domain inversion after noise filtering.

Comparing Figure 17b–d, when η = 0.5, the errors are mainly concentrated in the heating and cooling phases, with the heating phase being particularly significant. When η = 4, the errors are mainly concentrated in the constant temperature phase and some parts of the cooling phase. When η = 8, errors exist during the heating, constant temperature, and cooling phases. Therefore, it is necessary to adjust the noise filtering parameters in real time according to the changes in the response data. Adaptive noise filtering is required in practical applications. Comparing Figure 17c,e, Figure 17e shows the temperature calculated by full-time domain inversion based on the denoised global data. The results show that the appropriate noise filtering parameters and inversion window size can effectively solve the important problem of large error in the cooling stage during the full-time domain inversion, so as to achieve the accurate inversion of the temperature in the full-time domain.

The optimal window size coefficient calculated based on real measurement data is greater than the optimal value obtained from the numerical calculations (η = 1). In real measurement processes, various uncontrollable factors, such as measurement noise caused by electromagnetic interference and the effects of non-solid heat transfer, can reduce inversion accuracy. Therefore, more data, i.e., a larger window size, are needed to accurately invert the temperature. From the perspective of temperature sensors, replacing electrical sensors such as thermocouples with electromagnetic interference-resistant sensors like FBG sensors can reduce data noise and thus effectively decrease inversion errors. Although the real-time inversion method has a good generalizability, it is essentially based on the theory of solid heat transfer. Non-solid heat transfer within the material can reduce the accuracy of this method. During the heating of the TPS using the quartz lamp, radiative heat transfer plays a significant role in the initial heating stage. Therefore, the inversion calculation results generally exhibit overshoot. By gradually increasing η to reduce the proportion of radiative heat transfer, the inversion error can be decreased.

## 5. Comparison

A comparative analysis of different algorithms for the thermal information inversion of applicable thermal protection materials is conducted, and the findings are presented in Table 1. In this paper, the computational delay caused by the execution of the algorithm on a PC (AMD Ryzen 7) was about 0.151 s. Taking into account the response delay of thermocouples, the final delay for inverting the surface temperature of the TPS based on this method will be less than 1 s.

## 6. Conclusions

In this paper, the real-time implementation of a full-time domain inversion method TIM-AB is studied by simulation and experiments. The overlapping sliding window method can effectively achieve the real-time performance of the TIM-AB method. The impacts of moving window size, slide step, sampling frequency, and data noise on the real-time inversion accuracy are discussed. The conclusions of this study are summarized as follows.

(1) Numerical simulations analyzed the relationship between inversion accuracy and moving window size under Gaussian thermal shock. The results show the mean relative error of real-time inversion has an exponential relationship with the moving window size, with the optimal duration of window being 1 time that of the thermal hysteresis time. When the window slide step is smaller than the sensor’s data collection per unit time, the stack size scarcely affects inversion accuracy. For insulation materials with a large thermal hysteresis time, as long as the thermocouple sampling frequency exceeds 1 Hz, the influence of sampling frequency on the inversion accuracy is minimal, and the material’s surface temperature can be effectively retrieved.
(2) Experiments using a quartz lamp heater for lateral heating of the TPS verified that the real-time inversion method can accurately invert the surface temperature of the TPS materials using real data. Without noise filtering, the mean relative error of real-time inversion is 18.8%, higher than the 15.7% error of full-time domain inversion. Data noise from electromagnetic interference and other factors significantly can increase the inversion error.
(3) Three noise filtering methods, Gaussian, Savitzky-Golay, and moving average, are compared, with Savitzky-Golay proving the most effective. When the noise filtering window size is constant, the inversion error remains exponentially related to the moving window size. However, when the noise filtering window size varies, no monotonic relationship exists between the inversion error and moving window size. By adjusting the noise filtering window size, real-time inversion accuracy is significantly enhanced, reducing the mean relative error to 6.7%.

This paper presents a real-time inversion method for calculating the surface temperature of the TPS without requiring input on thermal properties. Its noise resistance, high speed, and accuracy make it a promising solution for various engineering applications, particular in the aerospace industry.

## Figures and Tables

**Table 1:** Different algorithms for thermal information inversion.

Reference	Key Approach for Solving IHCP	Features and Functions
[22]	Tikhonov regularization	Suitable for 1-D heat transfer of a multilayer medium; Robust noise resistance; Near real time, 17 s delay.
[24]	Tikhonov digital filter	Suitable for 1-D heat transfer of multilayer medium; Near real time.
[28]	Rapid computation combined with hybrid neural networks	Suitable for 2-D heat transfer; Based on algorithm pre-training; Real time, Second delay.
[34]	Sequential Function Specification and Truncated Singular Value Decomposition	Suitable for 2-D heat transfer; Stable thermal parameters required.
[35]	Tikhonov digital filter	Suitable for 1-D heat transfer; Near real time.
This paper	The Auto-Regression with eXtra and Overlapping Sliding Window	Suitable for 1-D heat transfer; No thermal parameters required; Robust noise resistance; Real time, less than 1 s delay.

## Data Availability

The original contributions presented in this study are included in the article. Further inquiries can be directed to the corresponding authors.

## Abbreviations

The following abbreviations are used in this manuscript:

TPS	Thermal Protection Structure
IHCP	Inverse Heat Conduction Problem
TIM-AB	Temperature Inversion Method with Adaptive Boundary
ARX	Auto-Regression with eXtra
FBG	Fiber Bragg Grating
TC	Thermocouple

## References

[1] Zhang S., Zhang Z., Wang Y., Zheng H. (2024). Design and performance verification of thermal protection structure of high temperature probe in aeroengine. Appl. Therm. Eng..

[2] Sapozhnikov S.Z., Mityakov V.Y., Mityakov A.V., Mozhaĭskiĭ S.A. (2008). Gradient-type sensors for heat flux measurements high temperatures. Tech. Phys. Lett..

[3] Trubachev S.A., Korobeinichev O.P., Shmakov A.G., Sagitov A.R. (2024). Method of heat flux measurement in solid fuel flames using semiconductor sensors. Combust. Explos. Shock Waves.

[4] Sakharov Valerii A., Popov Pavel A., Monakhov Nikolai A. (2023). Heat flux measurements of high speed flow around an axisymmetric body using sensors based on anisotropic thermoelements. St. Petersburg Polytech. Univ. J. Phys. Math..

[5] Le V.T., Ha S., Ha N., Goo N.S. (2021). Advanced sandwich structures for thermal protection systems in hypersonic vehicles: A review. Compos. Part B Eng..

[6] Wang F., Zhang P., Wang Y., Sun C., Xia X. (2022). Real-time identification of severe heat loads over external interface of lightweight thermal protection system. Therm. Sci. Eng. Prog..

[7] Nenarokomov A.V., Alifanov O.M., Budnik S.A., Netelev A.V. (2016). Research and development of heat flux sensor for ablative thermal protection of spacecrafts. Int. J. Heat Mass Transf..

[8] Tallman T.N., Frueh S., Lin C., Wertz J., Cherry M., Apostolov Z.D., Rueschhoff L.M. (2023). The electrical response of refractory carbon/carbon composites to high-temperature ablation: A pathway to embedded sensing in extreme environments. Compos. Part B Eng..

[9] Sobota T., Taler D., Taler J. (2024). Measurement of the temperature of the fluid inside the tube based on the temperature measurement of the thermally insulated outer surface. Int. J. Heat Mass Transf..

[10] Chen S., Su Z., Dai M., Xue C., Tao J., Hai Z. (2024). A General Super-Resolution Approach Integrating Physical Information for Temperature Field Measurement. Sensors.

[11] Oliveira A.V.S., Avrit A., Gradeck M. (2021). Thermocouple response time estimation and temperature signal correction for an accurate heat flux calculation in inverse heat conduction problems. Int. J. Heat Mass Transf..

[12] Mohebbi F. (2020). Explicit Sensitivity Coefficients for Estimation of Temperature-Dependent Thermophysical Properties in Inverse Transient Heat Conduction Problems. Computation.

[13] Kumar S., Mahulikar S.P. (2016). Reconstruction of aero-thermal heating and thermal protection material response of a Reusable Launch Vehicle using inverse method. Appl. Therm. Eng..

[14] Brociek R., Hetmaniok E., Słota D. (2023). Reconstruction of aerothermal heating for the thermal protection system of a reusable launch vehicle. Appl. Therm. Eng..

[15] Jiang X., Wang X., Wen Z., Wang H. (2024). Resolution-independent generative models based on operator learning for physics-constrained Bayesian inverse problems. Comput. Methods Appl. Mech. Eng..

[16] Gu J.h., Hong M., Yang Q.Q., Heng Y. (2023). A fast inversion approach for the identification of highly transient surface heat flux based on the generative adversarial network. Appl. Therm. Eng..

[17] Santos J.A., Oliveira J.R.F., do Nascimento J.G., Fernandes A.P., Guimaraes G. (2021). Simultaneous estimation of thermal properties via measurements using one active heating surface and Bayesian inference. Int. J. Therm. Sci..

[18] Tian J.M., Chen B., Zhou Z.F. (2017). Methodology of surface heat flux estimation for 2D multi-layer mediums. Int. J. Heat Mass Transf..

[19] Wu T., Zhang C., Ji H., Zhang Y., Tao C., Qiu J. (2023). Heat Flux Identification of Aircraft Structure with Artificial Neural Network Compensation. J. Thermophys. Heat Transf..

[20] Cebo-Rudnicka A., Malinowski Z. (2019). Identification of heat flux and heat transfer coefficient during water spray cooling of horizontal copper plate. Int. J. Therm. Sci..

[21] Samadi F., Woodbury K., Kowsary F. (2020). Optimal combinations of Tikhonov regularization orders for IHCPs. Int. J. Therm. Sci..

[22] Uyanna O., Najafi H., Rajendra B. (2021). An inverse method for real-time estimation of aerothermal heating for thermal protection systems of space vehicles. Int. J. Heat Mass Transf..

[23] Najafi H., Woodbury K.A., Beck J.V., Keltner N.R. (2015). Real-time heat flux measurement using directional flame thermometer. Appl. Therm. Eng..

[24] Najafi H., Woodbury K.A., Beck J.V. (2015). A filter based solution for inverse heat conduction problems in multi-layer mediums. Int. J. Heat Mass Transf..

[25] Cebula A., Taler J. (2014). Determination of transient temperature and heat flux on the surface of a reactor control rod based on temperature measurements at the interior points. Appl. Therm. Eng..

[26] Khatoon S., Phirani J., Bahga S.S. (2023). Fast Bayesian inference for inverse heat conduction problem using polynomial chaos and Karhunen–Loeve expansions. Appl. Therm. Eng..

[27] Qi H., Wen S., Wang Y.F., Ren Y.T., Wei L.Y., Ruan L.M. (2019). Real-time reconstruction of the time-dependent heat flux and temperature distribution in participating media by using the Kalman filtering technique. Appl. Therm. Eng..

[28] Zheng Y., Yin Z. (2025). Cutting temperature field online reconstruction using temporal convolution and deep learning networks. Int. J. Heat Mass Transf..

[29] Wan S., Wang K., Xu P., Huang Y. (2022). Numerical and experimental verification of the single neural adaptive PID real-time inverse method for solving inverse heat conduction problems. Int. J. Heat Mass Transf..

[30] Najafi H., Woodbury K.A. (2015). Online heat flux estimation using artificial neural network as a digital filter approach. Int. J. Heat Mass Transf..

[31] Chen Y., Chen Q., Ma H., Chen S., Fei Q. (2025). Transfer machine learning framework for efficient full-field temperature response reconstruction of thermal protection structures with limited measurement data. Int. J. Heat Mass Transf..

[32] Zhang L., Li L., Ju H., Zhu B. (2010). Inverse identification of interfacial heat transfer coefficient between the casting and metal mold using neural network. Energy Convers. Manag..

[33] He Z., Ni F., Wang W., Zhang J. (2021). A physics-informed deep learning method for solving direct and inverse heat conduction problems of materials. Mater. Today Comm..

[34] Nakamura T., Kamimura Y., Igawa H., Morino Y. (2014). Inverse analysis for transient thermal load identification and application to aerodynamic heating on atmospheric reentry capsule. Aerosp. Sci. Technol..

[35] Woodbury K.A., Beck J.V., Najafi H. (2014). Filter solution of inverse heat conduction problem using measured temperature history as remote boundary condition. Int. J. Heat Mass Transf..

[36] Huang W., Li J., Liu D. (2022). Real-Time Solution of Unsteady Inverse Heat Conduction Problem Based on Parameter-Adaptive PID with Improved Whale Optimization Algorithm. Energies.

[37] Najafi H., Woodbury K.A., Beck J.V. (2015). Real time solution for inverse heat conduction problems in a two-dimensional plate with multiple heat fluxes at the surface. Int. J. Heat Mass Transf..

[38] Deng S., Hwang Y. (2006). Applying neural networks to the solution of forward and inverse heat conduction problems. Int. J. Heat Mass Transf..

[39] Zhao X., Jin K.Z., Yan M.Y., Nan P.Y., Zhou F., Xin G.G., Lim K., Ahmad H., Zhang Y.P., Yang H.Z. (2025). Inverse heat transfer for real-time thermal evaluation of aircraft thermal protection structure with embedded FBG sensors. Appl. Therm. Eng..

[40] Kalberla P.M.W., Haud U. (2018). Properties of cold and warm HI gas phases derived from a Gaussian decomposition of HI4PI data. Astron. Astrophys..

[41] Li W., Sigmund O., Zhang X.S. (2024). Analytical realization of complex thermal meta-devices. Nat. Commun..

[42] Ali M.A., Shimoda M. (2022). Toward multiphysics multiscale concurrent topology optimization for lightweight structures with high heat conductivity and high stiffness using MATLAB. Struct. Multidiscip. Optim..

[43] Li W., Huang J., Zhang Z., Wang L., Huang H., Liang J. (2021). A model for thermal protection ablative material with local thermal non-equilibrium and thermal radiation mechanisms. Acta Astronaut..

[44] Feng J., Liu M., Ma S., Yang J., Mo W., Su X. (2020). Micro-nano scale heat transfer mechanisms for fumed silica based thermal insulating composite. Int. Commun. Heat Mass Transf..

[45] Tychanicz-Kwiecień M., Wilk J., Gil P. (2019). Review of high-temperature thermal insulation materials. J. Thermophys. Heat Transf..

